# Melatonin Attenuates Cisplatin-Induced Acute Kidney Injury through Dual Suppression of Apoptosis and Necroptosis

**DOI:** 10.3390/biology8030064

**Published:** 2019-08-30

**Authors:** Jong Woo Kim, Jungmin Jo, Jung-Yeon Kim, Misun Choe, Jaechan Leem, Jae-Hyung Park

**Affiliations:** 1 Department of Physiology, School of Medicine, Keimyung University, Daegu 42601, Korea; 2 Department of Hematology-Oncology, Inje University Seoul Paik Hospital, Seoul 04551, Korea; 3 Department of Immunology, School of Medicine, Catholic University of Daegu, Daegu 42472, Korea; 4 Department of Pathology, School of Medicine, Keimyung University, Daegu 42472, Korea

**Keywords:** melatonin, cisplatin, acute kidney injury, apoptosis, necroptosis

## Abstract

Melatonin is well known to modulate the sleep–wake cycle. Accumulating evidence suggests that melatonin also has favorable effects such as anti-oxidant and anti-inflammatory properties in numerous disease models. It has been reported that melatonin has therapeutic effects against cisplatin-induced acute kidney injury (AKI). However, mechanisms underlying the therapeutic action of melatonin on the renal side-effects of cisplatin therapy remain poorly understood. In this study, we showed that melatonin treatment significantly ameliorates cisplatin-induced acute renal failure and histopathological alterations. Increased expression of tubular injury markers was largely reduced by melatonin. Melatonin treatment inhibited caspase-3 activation and apoptotic cell death. Moreover, protein levels of key components of the molecular machinery for necroptosis were decreased by melatonin. Melatonin also attenuated nuclear factor-κB activation and suppressed expression of pro-inflammatory cytokines. Consistent with in vivo findings, melatonin dose-dependently decreased apoptosis and necroptosis in cisplatin-treated mouse renal tubular epithelial cells. Collectively, our findings suggest that melatonin ameliorates cisplatin-induced acute renal failure and structural damages through dual suppression of apoptosis and necroptosis. These results reveal a novel mechanism underlying the therapeutic effect of melatonin against cisplatin-induced AKI and strengthen the idea that melatonin might be a promising therapeutic agent for the renal side-effects of cisplatin therapy.

## 1. Introduction

Cisplatin is a platinum-based chemotherapy drug that has been used worldwide to treat various types of cancer. However, its clinical use is frequently limited by severe adverse reactions including nephrotoxicity and hepatotoxicity [1,2]. Previous studies showed that the kidneys accumulate more cisplatin than other organs and that the proximal tubules are principally damaged by cisplatin [3]. Approximately, 20% of patients treated with cisplatin suffer from nephrotoxicity such as acute kidney injury (AKI), while incidence of hepatotoxicity is lower [2,4]. Cisplatin-induced AKI is dose dependent and thereby limits the dose of drug that can be used [1]. In comparison, hepatotoxicity can occur only when cisplatin is administered at high doses [5]. Thus, cisplatin-induced AKI is considered one of the major side-effects of cisplatin therapy. Although some procedures including hydration management are used, there is still no specific pharmacotherapy for cisplatin-induced AKI.

The mechanism of cisplatin-induced AKI is complex and includes multiple cellular and molecular pathways [1,2]. Among them, renal tubular cell apoptosis has been recognized as a major contributor to the development of cisplatin-induced AKI. Moreover, accumulating evidence suggests that necroptosis, a form of regulated necrotic cell death, is also largely responsible for the renal side-effects of cisplatin therapy [6,7]. Thus, renal tubular cell death, including apoptosis and necroptosis, may represent a useful target for the prevention of cisplatin-induced AKI. Indeed, a recent study reported a synergistic protective effect of inhibitors of apoptosis and necroptosis against cisplatin-induced AKI [8].

Melatonin is a pineal hormone that regulates the sleep-wake cycle [9]. In addition to the role of melatonin in the modulation of circadian rhythm, it has been shown that melatonin exerts therapeutic effects against the renal side-effects of cisplatin therapy [10,11,12]. The beneficial effects of melatonin were associated with its anti-oxidant and anti-inflammatory properties. Recent studies also showed that melatonin has inhibitory effects on apoptosis and necroptosis in several disease models such as cardiac ischemia-reperfusion injury [13,14,15] and liver fibrosis [16,17]. However, the effect of the hormone on apoptosis and necroptosis in cisplatin-induced AKI has not yet been investigated.

In this study, we showed that melatonin treatment inhibits apoptosis and necroptosis in renal tubules of cisplatin-treated mice, resulting in amelioration of renal dysfunction and histological abnormalities. Cisplatin-induced apoptosis and necroptosis were also effectively suppressed by melatonin in mouse renal tubular epithelial (TCMK-1) cells. These results reveal a novel mechanism underlying the therapeutic effect of the hormone against cisplatin-induced AKI.

## 2. Materials and Methods

### 2.1. Animals and Drug Treatment

Seven-week-old male C57BL/6N mice (Hanasangsa, Busan, South Korea) were adapted to the facility for one week before study. The mice were randomly grouped into the following groups (n = 10 for each group): Control (Con), cisplatin alone (CP), and melatonin in combination with cisplatin (CP+MEL). For cisplatin treatment, mice received an intraperitoneal injection with 15 mg/kg cisplatin (dissolved in 0.9% normal saline). Control mice were administered intraperitoneally with the equal volume of the vehicle. To evaluate the potential effects of melatonin (Sigma-Aldrich, St. Louis, MO, USA) on the renal side-effects of cisplatin therapy, mice were injected intraperitoneally with 20 mg/kg melatonin for 3 days before and 2 days after cisplatin treatment. Mice were sacrificed 72 h after cisplatin injection. The doses of cisplatin [18,19] and melatonin [20,21] were determined based on previous reports. All animal experiments were approved by the Keimyung University Institutional Ethics Committee (KM-2017-40R1).

### 2.2. Cell Culture and Treatments

TCMK-1 cells were obtained from the Korean Cell Line Bank (Seoul, South Korea) and cultured in Dulbecco’s modified Eagle Medium (DMEM) containing 10% fetal bovine serum and maintained in a humidified incubator at 37 °C under 5% CO₂ and 95% air. The cells were pretreated with 1 mM melatonin for 1 h and then treated with 2 μg/mL cisplatin for 24 h.

### 2.3. Renal Function

Serum levels of blood urea nitrogen (BUN) and creatinine were analyzed using commercial kits (Bioassay Systems, Hayward, CA, USA) according to the manufacturer’s instructions.

### 2.4. Histological and Immunohistochemical Staining

Kidney tissues obtained from mice were fixed in 4% phosphate-buffered paraformaldehyde and embedded in paraffin. The paraffin blocks were cut into thin sections and probed with hematoxylin and eosin (H&E) stain and periodic acid-Schiff (PAS) stain. The degree of tubular injury was scored as previously described [18]. For immunohistochemistry, the kidney sections were probed with antibodies against neutrophil gelatinase-associated lipocalin (NGAL; Abcam, Cambridge, MA, USA) or kidney injury molecule-1 (Kim-1; Sigma-Aldrich).

### 2.5. TdT-Mediated dUTP Nick End Labeling (TUNEL) Assay

Apoptosis was determined in the kidney sections using a commercial kit (Roche Diagnostics, Indianapolis, IN, USA) according to the manufacturer’s instruction. Briefly, the sections were deparaffinized in xylene, rehydrated using descending grades of ethanol, and permeabilized for 30 min at room temperature with proteinase K in 10 mM Tris-HCl, pH 7.4–8. After washing, the sections were incubated in the TUNEL reaction mixture for 1 h at 37 °C. Nuclei were counterstained with DAPI. Images were captured using the NIKON A1+ confocal microscope. TUNEL-stained apoptotic cells were counted in five randomly chosen fields (×200 magnification) per each kidney. In vitro, five randomly chosen fields (×50 magnification) were captured in each group. Apoptotic percentage was calculated as the ratio of TUNEL-stained nuclei to the total cell nuclei counterstained by DAPI.

### 2.6. Lactate Dehydrogenase (LDH) Release Assay

Activity of LDH released into the culture medium was analyzed using a commercial kit (Sigma-Aldrich) according to the manufacturer’s instruction.

### 2.7. Western Blot Analysis

Equal amounts of protein from each sample were resolved by SDS-PAGE, and then transferred onto a nitrocellulose membrane. After blocking, the membrane was incubated with the following primary antibodies: Anti-poly(ADP-ribose) polymerase 1 (PARP1; Abcam), anti-cleaved caspase-3 (Cell Signaling, Danvers, MA, USA), anti-receptor-interacting serine/threonine protein kinase 1 (RIPK1; Cell Signaling), anti-RIPK3 (Cell Signaling), anti-nuclear factor-κB (NF-κB) p65 (Sigma-Aldrich), anti-phospho-NK-κB p65 (Sigma-Aldrich), anti-Kim-1 (Sigma-Aldrich) and anti-glyceraldehyde-3-phosphate dehydrogenase (GAPDH; Cell Signaling) antibody. Then, the membrane was incubated with horseradish peroxidase-conjugated secondary antibodies, and signals were detected using an enhanced chemiluminescence detection system (Thermo Fisher Scientific, Waltham, MA, USA). The protein expression level was normalized against GAPDH.

### 2.8. Gene Expression Analysis 

Total RNA was extracted from kidney tissues using TRIzol Reagent (Sigma-Aldrich), and then reverse transcribed into cDNA using 5X first-strand buffer (Invitrogen, Carlsbad, CA, USA), dNTP Mix (Promega, Madison, WI, USA), oligo(dT) primers (Macrogen, Seoul, South Korea), a reverse transcriptase enzyme (Invitrogen), and a ribonuclease inhibitor (Invitrogen). Quantitative real-time reverse transcription polymerase chain reaction (RT-PCR) was performed using the Real-Time PCR 7500 system (Applied Biosystems, Foster City, CA, USA) and Power SYBR Green PCR Master Mix (Applied Biosystems). Primer sequences used are listed in Table 1. Ribosomal protein L32 was used to normalize the expression levels of the other genes.

### 2.9. Statistical Analysis

Data are expressed as mean ± SEM. SPSS version 20.0 (SPSS, Chicago, IL, USA) was used for statistical analyses. The differences between the 3 groups were analyzed using ANOVA followed by post-hoc Bonferroni multiple comparison test. A *P*-value ˂ 0.05 was considered statistically significant.

## 3. Results

### 3.1. Melatonin Ameliorated Cisplatin-Induced AKI

**Figure 1:**
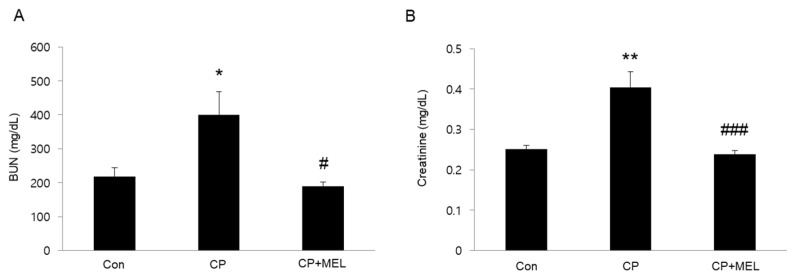
Effects of melatonin (MEL) on renal function in mice treated with cisplatin (CP). (**A**) Serum levels of blood urea nitrogen (BUN). (**B**) Serum creatinine levels. * *P* < 0.05 and ** *P* < 0.01 vs. Con. ^#^ *P* < 0.05 and ^###^ *P* < 0.001 vs. CP.

**Figure 2:**
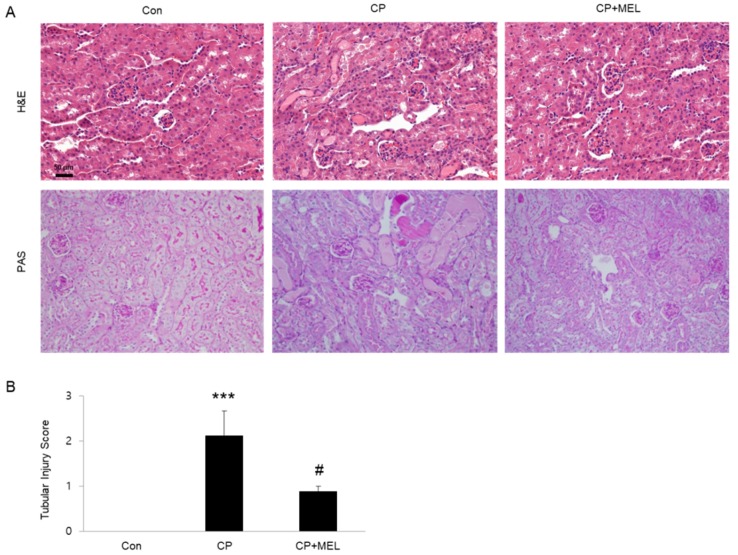
Effects of melatonin (MEL) on renal histology in mice treated with cisplatin (CP). (**A**) Representative images of hematoxylin and eosin (H&E) and periodic acid-Schiff (PAS) staining. Scale bar: 50 μm. (**B**) Tubular injury score. *** *P* < 0.001 vs. Con. ^#^ *P* < 0.05 vs. CP.

To induce AKI, mice were intraperitoneally injected with cisplatin (15 mg/kg). Administration of melatonin significantly attenuated acute renal failure, as assessed by reduced blood urea nitrogen (BUN) and creatinine levels, at 3 days after cisplatin injection (Figure 1A,B). Histological staining also showed that melatonin treatment significantly ameliorated cisplatin-associated histopathological alterations such as tubular dilatation, tubular atrophy and cast formation (Figure 2A,B).

**Figure 3:**
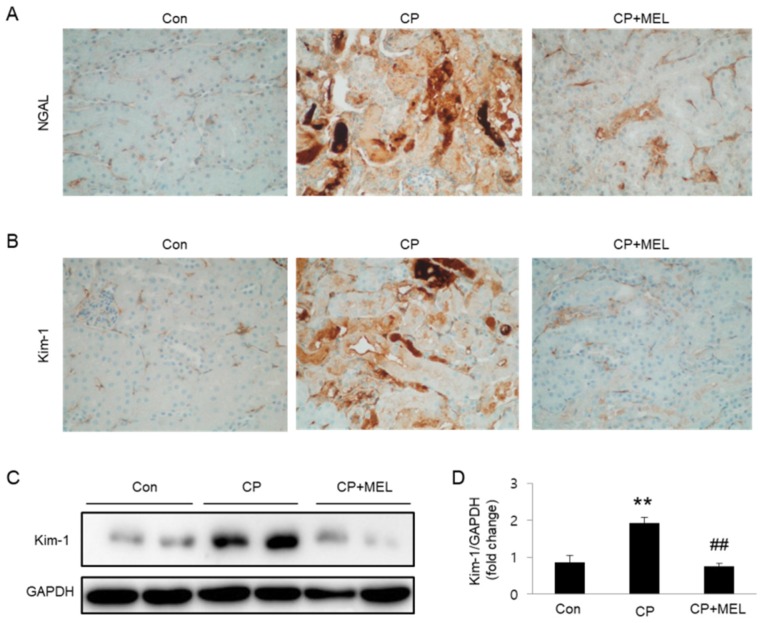
Effects of melatonin (MEL) on expression of tubular injury markers in mice treated with cisplatin (CP). Representative images of immunohistochemical staining using anti-neutrophil gelatinase-associated lipocalin (NGAL) (**A**) or anti-kidney injury molecule-1 (Kim-1) antibody (**B**). Scale bar: 25 μm. (**C**) Western blots of Kim-1 level in kidneys. (**D**) Quantification of Kim-1 level. ** *P* < 0.01 vs. Con. ^##^ *P* < 0.01 vs. CP.

To further investigate the effect of melatonin in tubular damage, we evaluated the levels of tubular injury markers in kidneys. Immunohistochemical staining revealed that expression level of NGAL and Kim-1 was largely elevated in damaged tubules of mice treated with cisplatin alone and these changes were largely reversed by melatonin (Figure 3A,B). Consistently, melatonin treatment also markedly reversed cisplatin-induced elevation in Kim-1 protein level in kidneys (Figure 3C,D).

### 3.2. Melatonin Suppressed Tubular Cell Apoptosis in Kidneys of Mice Treated with Cisplatin

**Figure 4:**
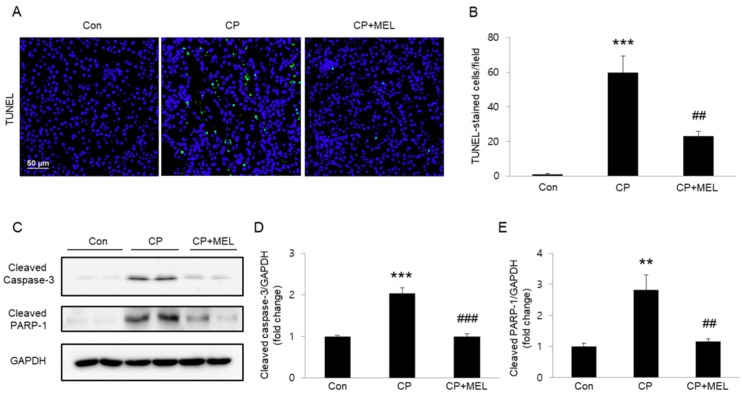
Effects of melatonin (MEL) on apoptotic cell death in kidneys of mice treated with cisplatin (CP). (**A**) Representative images of TdT-mediated dUTP nick end labeling (TUNEL) staining. Scale bar: 50 μm. (**B**) Number of TUNEL-positive cells. (**C**) Western blots of cleaved caspase-3 and cleaved poly(ADP-ribose) polymerase 1 (PARP-1) levels in kidneys. (**D**) Quantification of cleaved caspase-3 level. (**E**) Quantification of cleaved PARP-1 level. ** *P* < 0.01 and *** *P* < 0.001 vs. Con. ^##^ *P* < 0.01 and ^###^ *P* < 0.001 vs. CP.

To reveal the underlying mechanisms for therapeutic effects of melatonin against the renal side-effects of cisplatin therapy, TUNEL staining of the kidney sections was performed to identify apoptotic cells. Administration of melatonin significantly reversed an increase in the number of TUNEL-stained cells after cisplatin injection (Figure 4A,B). Moreover, melatonin treatment also largely reversed cisplatin-associated elevation in protein levels of activated caspase-3 and cleaved PARP-1 in kidneys (Figure 4C–E).

### 3.3. Melatonin Suppressed Tubular Cell Necroptosis in Kidneys of Mice Treated with Cisplatin

**Figure 5:**
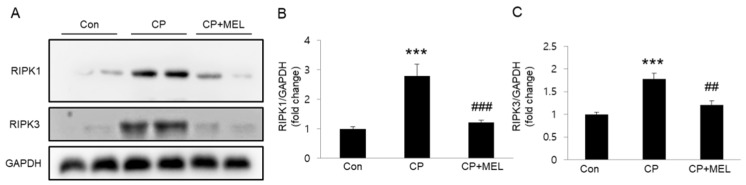
Effects of melatonin (MEL) on necroptosis in kidneys of mice treated with cisplatin (CP). (**A**) Western blots of receptor-interacting serine/threonine protein kinase 1 (RIPK1) and RIPK3 levels in kidneys. (**B**) Quantification of RIPK1 level. (**C**) Quantification of RIPK3 level. *** *P* < 0.001 vs. Con. ^##^ *P* < 0.01 and ^###^ *P* < 0.001 vs. CP.

Recent studies suggest that in addition to apoptosis, necroptosis also is critically involved in the renal side-effects of cisplatin therapy [6,7]. To explore the effect of melatonin on cisplatin-associated necroptosis, we measured protein levels of RIPK1 and RIPK3 in kidneys. We observed that cisplatin treatment significantly increased their protein levels in kidneys and these changes were largely attenuated by melatonin (Figure 5A–C), suggesting that melatonin inhibits cisplatin-induced necroptosis.

### 3.4. Melatonin Attenuated Inflammatory Responses in Kidneys of Mice Treated with Cisplatin

**Figure 6:**
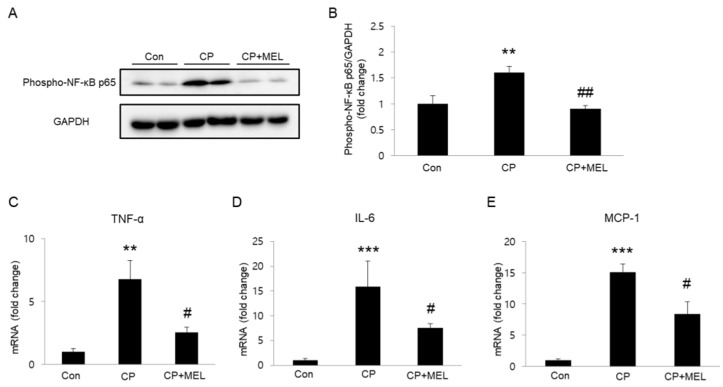
Effects of melatonin (MEL) on renal inflammation in mice treated with cisplatin (CP). (**A**) Western blots of phospho-nuclear factor-κB (NF-κB) p65 level in kidneys. (**B**) Quantification of phospho-NF-κB p65 level. Real-time reverse transcription polymerase chain reaction (RT-PCR) analysis of tumor necrosis factor-α (TNF-α) (**C**), interleukin-6 (IL-6) (**D**), and monocyte chemoattractant protein-1 (MCP-1) (**E**) in kidneys. ** *P* < 0.01 and *** *P* < 0.001 vs. Con. ^#^ *P* < 0.05 and ^##^ *P* < 0.01 vs. CP.

Plasma membrane rupture in necroptosis results in release of intracellular components that stimulate immune system and inflammation [22]. Given that cisplatin induces NF-κB activation and subsequently promotes expression of pro-inflammatory cytokines, we next investigated the effect of melatonin on NF-κB phosphorylation in kidneys. We found that administration of melatonin markedly reversed elevated protein level of phospho-NF-κB in kidneys (Figure 6A,B). In addition, mRNA expression of tumor necrosis factor-α (TNF-α), interleukin-6 (IL-6), and monocyte chemoattractant protein-1 (MCP-1) was also significantly reduced by melatonin (Figure 6C–E).

### 3.5. Melatonin Suppressed Cisplatin-Induced Apoptosis and Necroptosis In Vitro

**Figure 7:**
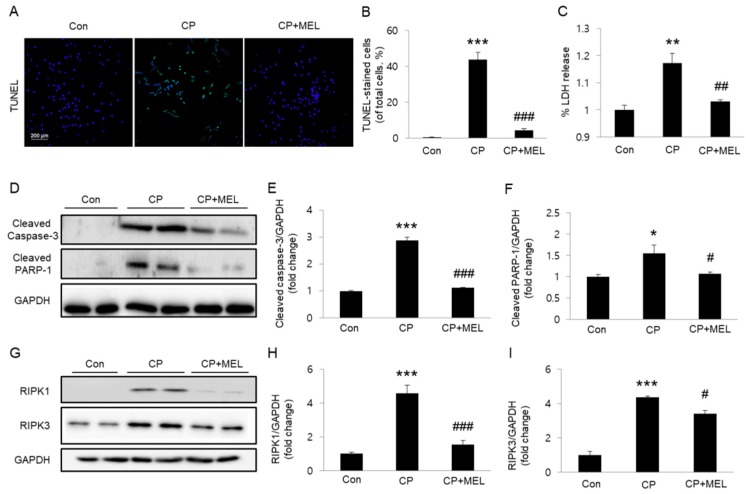
Effects of melatonin (MEL) on cisplatin (CP)-induced apoptosis and necroptosis in cultured TCMK-1 cells. TCMK-1 cells were pretreated with 1 mM MEL for 1 h and then treated with 2 μg/mL CP for 24 h. (**A**) Representative images of TdT-mediated dUTP nick end labeling (TUNEL) staining. Scale bar: 200 μm. (**B**) Number of TUNEL-positive cells. (**C**) lactate dehydrogenase (LDH) release assay. (**D**) Western blots of cleaved caspase-3 and cleaved poly(ADP-ribose) polymerase 1 (PARP-1) levels. (**E**) Quantification of cleaved caspase-3 level. (**F**) Quantification of cleaved PARP-1 level. (**G**) Western blots of receptor-interacting serine/threonine protein kinase 1 (RIPK1) and RIPK3 levels. (**H**) Quantification of RIPK1 level. (**I**) Quantification of RIPK3 level. *P < 0.05, ** *P* < 0.01, and ***P < 0.001 vs. Con. ^#^ *P* < 0.05, ^##^ *P* < 0.01, and ^###^ *P* < 0.001 vs. CP.

We next examined whether melatonin could also inhibit cisplatin-induced cell death in cultured TCMK-1 cells. We found that pretreatment with melatonin significantly reversed an increase in the number of TUNEL-stained cells after cisplatin treatment (Figure 7A,B). LDH release assay also showed that melatonin significantly reversed cisplatin-induced LDH release (Figure 7C). In addition, elevated protein expression of activated caspase-3 and cleaved PARP-1 after cisplatin treatment was greatly attenuated by melatonin pretreatment (Figure 7D–F). Pretreatment with melatonin also significantly reversed elevated protein expression of RIPK1 and RIPK3 in cisplatin-treated cells (Figure 7G–I).

## 4. Discussion

In this study, we showed that administration of melatonin significantly inhibited apoptosis and necroptosis in renal tubules of cisplatin-treated mice. These effects may contribute to amelioration of cisplatin-induced renal dysfunction and histological abnormalities. Consistent with in vivo data, cisplatin-induced apoptosis and necroptosis in TCMK-1 cells were also effectively suppressed by melatonin. Our findings provide a novel mechanism underlying the therapeutic effect of melatonin against cisplatin-induced AKI.

AKI is the most important dose-limiting side-effects of cisplatin-based chemotherapy. Thus, development of effective pharmacotherapy for cisplatin-induced AKI can have a strong clinical impact. In this study, we found that melatonin treatment significantly ameliorates acute renal failure in cisplatin-treated mice. Histopathological alterations were also significantly attenuated by melatonin. Because cisplatin has been known to induce severe tubular injuries in kidneys [23,24], we further examined the effect of melatonin on expression of tubular injury markers. Elevated levels of NGAL and Kim-1 in kidneys were found to be reduced by melatonin, indicating the therapeutic effect of melatonin against cisplatin-associated tubular injury. Collectively, these results suggest that melatonin ameliorates cisplatin-associated renal dysfunction and histological abnormalities in mice.

Tubular cell apoptosis is recognized as a critical process in the renal side-effects of cisplatin therapy [1,2]. Cisplatin can induce activation of the pro-apoptotic proteins, which form pores in the outer membrane of mitochondria, resulting in induction of cytochrome c release into cytosol and resultant activation of executioner caspases such as caspase-3. Previous studies have shown that in addition to its anti-oxidant and radical-scavenging properties, melatonin exerts an anti-apoptotic effect on various types of cells [13,25]. In this study, we found that melatonin greatly inhibited caspase-3 activation and subsequent cleavage of PARP-1 in kidneys after cisplatin injection. Furthermore, TUNEL staining confirmed the anti-apoptotic effect of melatonin. We also found that melatonin significantly suppressed cisplatin-induced apoptosis in vitro. Collectively, these findings indicate that melatonin has an anti-apoptotic effect in renal tubular cell apoptosis induced by cisplatin.

Although necrosis was originally considered to be an unprogrammed type of cell death, necroptosis has been suggested as a form of programmed necrosis [26]. It has been shown that a multiprotein complex composed of RIPK1 and RIPK3 plays a key regulatory role in initiating necroptosis [22]. Caspase-8 mediates apoptosis by activating downstream executioner caspases. When caspase-8 activity is suppressed, RIPK1 induces necroptosis by interacting with RIPK3, which regulates the phosphorylation of mixed lineage kinase domain-like protein (MLKL). This post-translational modification induces oligomerization and translocation of MLKL to the plasma membrane, where it disrupts membrane integrity, resulting in cell lysis. Rupture of plasma membrane leads to spilling of the intracellular contents, resulting in triggering inflammation. Indeed, recent studies suggest that necroptosis is an important pathogenic process in many inflammatory diseases [27,28]. Necroptosis was also shown to be critically involved in the renal side-effects of cisplatin therapy [6,7]. Expression of RIPK1 and RIPK3 was found to be increased in renal tubules after cisplatin injection [7]. In this study, we confirmed the increased levels of RIPK1 and RIPK3 in kidneys of cisplatin-treated mice. Interestingly, we found that melatonin treatment largely reversed their increase. We also performed LDH release assay to examine the effect of melatonin on cisplatin-associated necroptosis in tubular epithelial cells. We showed that melatonin dose-dependently reduced cisplatin-induced LDH release in cultured TCMK-1 cells. Cisplatin-induced upregulation of RIPK1 and RIPK3 was also significantly attenuated by melatonin. In agreement with our findings, melatonin was previously reported to prevent cardiac ischemia-reperfusion injury through suppression of RIPK3-dependent necroptosis [14,15]. In addition, melatonin ameliorated carbon tetrachloride-induced liver fibrosis by suppressing necroptosis [17]. In this study, we also observed that melatonin significantly attenuated NF-κB activation and renal levels of pro-inflammatory cytokines. These anti-inflammatory actions of melatonin may be attributed, at least in part, to its ability to inhibit necroptosis. Collectively, these findings indicate that melatonin inhibits cisplatin-induced necroptosis in tubular epithelial cells.

## 5. Conclusions

In conclusion, we demonstrate that melatonin inhibits apoptosis and necroptosis in cisplatin-induced AKI in vivo and in vitro. Given that both types of cell death play critical roles in cisplatin-induced AKI, these beneficial effects of melatonin are mainly responsible for its therapeutic action against cisplatin-induced renal dysfunction and structural damage. Although future studies will be needed to elucidate more details of the molecular mechanisms of melatonin’s suppressive effects on apoptosis and necroptosis, our findings reveal a novel mechanism underlying the therapeutic effect of melatonin against the renal side-effects of cisplatin therapy. These results strengthen the idea that melatonin may be a useful therapeutic agent for preventing cisplatin-induced AKI.

## Figures and Tables

**Table 1:** Primers used for quantitative real-time reverse transcription polymerase chain reaction (RT-PCR).

Gene	Primer Sequence (5’→3’)	Product Size (bp)
*TNF-α*	Forward: CCCTCACACTCAGATCATCTTCT Reverse: GCTACGACGTGGGCTACAG	61
*IL-6*	Forward: TAGTCCTTCCTACCCCAATTTCC Reverse: TTGGTCCTTAGCCACTCCTTC	76
*MCP-1*	Forward: TTAAAAACCTGGATCGGAACCAA Reverse: GCATTAGCTTCAGATTTACGGGT	121
*L32*	Forward: ACATTTGCCCTGAATGTGGTReverse: ATCCTCTTGCCCTGATCCTT	199

TNF-α, tumor necrosis factor-α; IL-6, interleukin-6; MCP-1, monocyte chemoattractant protein-1.
